# A Stakeholder‐Informed Approach to the Development and Implementation of National Digital Registry and Learning Healthcare System (LHS) for Psychosis: Findings From the EPICare Platform

**DOI:** 10.1111/eip.70264

**Published:** 2026-09-21

**Authors:** Rachel Upthegrove, Melanie Lafanechere, Megan A. Pope, India Francis‐Crossley, John Ainsworth, Sophie M. Allan, Tumelo Bogatsu, Niyah Campbell, Richard J. Drake, Sonia Johnson, Mohammad Zia Ul Haq Katshu, Graham K. Murray, Sarah A. Sullivan, Justin Waring, Pauline Whelan, Sian L. Griffiths, James B. Kirkbride

**Affiliations:** ^1^ Department of Psychiatry University of Oxford Oxford UK; ^2^ Institute for Mental Health University of Birmingham Birmingham UK; ^3^ Division of Psychiatry UCL London UK; ^4^ Faculty of Biology, Medicine and Health, Division of Informatics, Imaging and Data Science, School of Health Sciences The University of Manchester Manchester UK; ^5^ Department of Clinical Psychology and Psychotherapies University of East Anglia Norwich UK; ^6^ Division of Psychology and Mental Health The University of Manchester Manchester UK; ^7^ Greater Manchester Mental Health NHS Foundation Trust Manchester UK; ^8^ North London Foundation Trust London UK; ^9^ Institute of Mental Health University of Nottingham Nottingham UK; ^10^ Nottinghamshire Healthcare NHS Foundation Trust Nottingham UK; ^11^ Department of Psychiatry University of Cambridge Cambridge UK; ^12^ Cambridgeshire and Peterborough Foundation Trust Cambridge UK; ^13^ Bristol Medical School, University of Bristol Bristol UK; ^14^ Health Services Management Centre University of Birmingham Birmingham UK; ^15^ School of Social Sciences and Humanities Loughborough University Loughborough UK; ^16^ Division of Informatics, Imaging and Data Sciences The University of Manchester Manchester UK

**Keywords:** co‐design, early intervention in psychosis, early psychosis, EIP, first episode psychosis, health informatics, health registry

## Abstract

**Introduction:**

Early Intervention in Psychosis services were established throughout England over two decades ago, yet despite aiming to optimise care, less than 40% of patients achieve full clinical recovery and notable inequities persist in accessing NICE‐recommended treatments. Fragmented electronic health record systems and limited systematic data capture conceal how disparities in care delivery and effectiveness affect recovery and remission. The Early Psychosis Informatics into Care, EPICare, programme aims to develop a national, learning healthcare system incorporating a standardised core dataset and clinician dashboard to support equitable, data‐driven care.

**Methods:**

Our stakeholder co‐design approach comprised three interlinked work packages delivered between 2021 and 2022. Using participatory co‐design, we conducted four multi‐stakeholder workshops and preparatory lived experience sessions with people with lived experience, clinicians, informaticians, academics, and policymakers. We identified essential data elements using adapted “MoSCoW” card sort methodology, refined content, and developed dashboard requirements. Parallel informatics and implementation research assessed technical feasibility, data maturity, and harmonisation requirements and evaluated anticipated barriers and facilitators to future adoption.

**Results:**

Consensus was achieved on core EPICare dataset “must haves” including sociodemographic variables, routine treatment interventions, Patient‐ and Clinician‐Reported Outcome Measures, and longitudinal symptom data. Stakeholders defined key dashboard features, prioritising usability and alignment with clinical workflow. Substantial variation in Trust‐level data infrastructure informed flexible technical architecture to mitigate implementation risk.

**Conclusion:**

EPICare stakeholder engagement delivered the blueprint for a co‐designed, technically feasible and nationally scalable specification for a psychosis learning healthcare system, enabling progression to platform development and piloting across five Early Intervention in Psychosis services in the NHS.

## 1 Introduction

Psychotic disorders are accompanied by substantial personal, family, societal, and carer burden. Psychotic disorders are unequally distributed throughout the population (Jongsma et al. 2019; Kirkbride et al. 2012; March et al. 2008), with the highest rates found in areas of deprivation (March et al. 2008; O'Donoghue et al. 2021), younger populations (Kirkbride et al. 2012), and people from minority ethnic backgrounds (Kirkbride et al. 2012). In England, Early Intervention in Psychosis (EIP) services are nationally commissioned to provide evidence‐based, multidisciplinary care according to eight national “Access and Waiting Time” standards for people experiencing first‐episode psychosis (FEP), introduced by the National Institute for Health and Care Excellence (NICE) and NHS England in 2016 (NICE 2014). These include a 14‐day maximum waiting time for initial treatment after eligible referral, and the offer of seven evidence‐based interventions (Anderson et al. 2018; Bird et al. 2010; Correll et al. 2018; NICE 2014) including cognitive behaviour therapy (CBT) for psychosis, family interventions, clozapine for people with inadequate clinical response to two antipsychotics, supported employment and education, annual physical health assessments, physical health interventions, and carer psychoeducation and support. Importantly, EIP care is cost‐effective relative to other forms of care and management for people with psychosis (McCrone et al. 2010), and EIP services are highly valued by service users (Lester et al. 2011).

Despite this strong evidence base for EIP services, only 30%–40% of people experiencing a first episode of psychosis (FEP) make a full clinical recovery (Catalan et al. 2021; Lally et al. 2017). Longer term outcomes also remain poor, with increased rates of physical illnesses (Upthegrove et al. 2013) and a reduced life expectancy of around 15 years compared with people who do not go on to develop severe mental illness (SMI) (Hjorthøj et al. 2017). Moreover, there is evidence of large variations in care (Fisher et al. 2008; Marwaha et al. 2016; Stokes et al. 2020; Upthegrove et al. 2013). Recent data indicates that people with psychosis from Black African and Caribbean backgrounds were 15%–30% less likely to receive CBT for psychosis than White British patients (Morris et al. 2020; Schlief et al. 2023), and 44% less likely to be offered clozapine (Das‐Munshi et al. 2018). Additionally, disparities persist in outcomes following EIP care, with social deprivation related to higher rates of relapse and need for continuing care in secondary mental health services (Kam et al. 2015; Puntis et al. 2018).

These individual and geographical inequities in EIP care for psychosis may also lead to disparities in downstream outcomes following psychosis, including symptomatic remission, functional recovery, and other outcomes highly valued by patients including time use, social connectedness, and a return to activities of daily living. Unfortunately, there is a paucity of evidence on this issue in England, or indeed worldwide. In England, our ability to understand the real‐world effectiveness of NICE‐recommended treatments for FEP (themselves primarily based on Randomised Control Trial evidence (NHS England 2023) which may not be representative of real world FEP samples) is severely hampered by the current ways in which routine health data in NHS EIP services are collated, processed, analysed and disseminated for clinical improvement and public mental health.

Several key issues inhibit improvement in this space. First, mental health care, including EIP service delivery in the NHS in England, is highly fragmented, with over 70 separate Mental Health Trusts (NHS provider organisations mandated with delivering mental health care in geographically defined population catchment areas). While there is standardisation in the delivery of EIP services within England, NHS Trusts can all implement service delivery, care models, IT systems, health governance legislation, and human resource management in different ways. For example, while over 70% of all NHS Mental Health Trusts in England use an electronic health record (EHR) system known as RiO, local configuration of these systems can make harmonisation of standardised EHR data challenging. Second, some routine health data is still collected via pen and paper, which is inefficient and unreliable. Third, although NHS England manages a centralised, unified dataset for mental health care in England (the Mental Health Services Dataset (MHSDS)), substantial challenges in its interoperability and data quality inhibit its practical use for providing timely, data‐driven insights into optimal individual clinical care, disparities in care provision, or understanding the real‐world effectiveness of treatments on patient outcomes. It was primarily established for service audit, performance evaluation, and economic costing purposes rather than informing clinical or public health care provision.

These issues and limitations are recognised by the national government and the NHS. For example, the new 10‐year health plan for the NHS, “Fit for the future”, was published in 2025 on the basis of three central pillars for effective NHS reform: analogue to digital, sickness to prevention, and hospital to community (Department of Health and Social Care and Prime Minister's Office 2025). Establishing a national learning healthcare system (LHS) in EIP care that collects representative, real‐time data on treatment and outcomes available at the service, team and individual levels could transform patient care and outcomes for psychosis under this national ambition. Such a platform could provide actionable real time insights to reduce disparities in care, improve digital data collection in psychosis care, and identify what worked for whom at scale. Clinically and cost‐effective secondary prevention strategies could be developed with representative data for care nationally, which have the potential to improve recovery and remission and prevent hospital admission.

Digital registries and LHS have been deployed successfully in the UK for other disease areas such as stroke, cystic fibrosis (CF), cancer and dementia (Heuschmann et al. 2008; Kelsey et al. 2020; Stephan et al. 2020; Taylor‐Robinson et al. 2018), transforming patient care and outcomes by identifying previously untreated risk factors and providing targeted interventions based on patient need (Langhorne et al. 2017; Mazo et al. 2020). We hope for the same parity of investment, innovation and action for psychosis.

To initiate this ambition, we developed a three‐stage programme to deliver an evidence‐based psychosis platform, capable of being implemented nationally, that would include a core data set of standardised measures that are meaningful to essential stakeholder groups (patients, informal carers, clinical teams, commissioners and policymakers) and an integrated clinical dashboard to inform clinical and patient care in real time. Our co‐developed solution would provide standardised information to understand the treated burden of psychosis in the NHS, evaluate whether treatment offered improves patient outcomes in real‐world contexts, identify priority areas for service improvement, and ensure equitable, responsive, local resource allocation. In this first stage, we aimed to co‐design and produce a framework and protocols for onward building, implementation, piloting, and evaluation of this national psychosis LHS.

## 2 Materials and Methods

We have previously reported the protocol for the Early Psychosis Informatics into Care (EPICare) stakeholder co‐design phase (Griffiths et al. 2024). In brief, a multi‐stakeholder Programme Management Group was established to oversee and coordinate three concurrent work packages over 12 months from November 2021 to the end of October 2022. The EPICare platform was reviewed and granted full approval by the Health Research Authority on November 8, 2021 (Ref: 306234). From the outset, we designed the EPICare platform to consist of two major components: the EPICare registry for psychosis to include a standardised set of measures to be collected routinely in EIP care, and the EPICare clinical dashboard, which we developed to provide clinicians with an easy‐to‐use, accessible tool that tracked patient progress on core measures during their EIP care in real time. Both elements of the EPICare platform were co‐designed with key stakeholders involved in receiving and delivering EIP care in England.

To facilitate effective co‐design, our stakeholder engagement methodology comprised three interrelated work packages, described in brief below, with further details in Supplemental Methods.

### 2.1 Work Package 1 (WP1): Stakeholder Co‐Design of the EPICare Platform Core Dataset and Clinician Dashboard

Using a participatory co‐design framework, we recruited a diverse group of stakeholders with expertise in early intervention for psychosis, including clinicians, academics, policymakers, and patient and public involvement (PPIE) representatives (recruitment process detailed in Supplement). The PPIE stakeholders were people with lived experience of using services for psychosis and/or supporting family members who use the services.

Four stakeholder workshops were convened online (between December 2021 and August 2022) in a facilitator‐led, semi‐structured format, including presentations, whole‐group discussions, and themed breakout sessions with both mixed group (random allocation) and streamed (by broad stakeholder type) group sessions on a per‐task basis. Essential materials were circulated to stakeholders in advance of each meeting. We convened additional online preparatory and debrief sessions for PPIE stakeholders, led by our PPIE coordinator, to provide ground‐setting and aid their understanding of the project and facilitate active participation in the main workshops. These methods intended to minimise the impact of traditional group identities and power structures during stakeholder workshops. Workshops and preparatory sessions were held online due to COVID‐19 restrictions.

Over the course of four workshops, stakeholders contributed to the development of a consensus framework. In Workshop 1, stakeholders reviewed a longlist of features initially identified by the Programme Management Group, prioritising essential and desirable data items for potential inclusion in both the EPICare core dataset and clinician dashboard across four domains (sociodemographic measures, treatment measures, Patient Reported Outcome Measures (PROMs), and Clinician Rated Outcome Measures (CROMs)) and providing written feedback to refine consensus. Workshop 2 focused on reprioritising these items using a card‐sort task, which aimed to identify the essential elements for inclusion under a hypothesised scenario allowing for data collection within a maximum 30‐min clinical timeframe. In Workshop 3, stakeholders identified the clinically important notifications, reminders, and features of the clinician dashboard, and determined their timing and frequency. In this workshop, we also sought to synthesise the findings from previous workshops via a modified Delphi approach to agree a final set of data priorities using an adapted version of the MoSCoW prioritisation method. MoSCoW prioritisation involves reaching a consensus between participants on which features of an intervention are “Must haves”, “Should haves”, “Could haves”, and “Won't haves”. Our adapted version categorised features of the EPICare platform into “Must have”, “Could have”, and “Won't have” sets. “Should have” features were evaluated and placed into “Must have” or “Could have” categories for implementation and communication purposes: for example, “Should have” features that were not required for the platform to operate but which met consensus for inclusion by stakeholders were coded as “Must haves”. Finally, Workshop 4 presented the refined framework, gathered feedback, and outlined the next steps for the development of the EPICare platform.

### 2.2 WP2: Informatics Architecture, Infrastructure, and Integration: Framework, Protocol and Toolkit

To minimise technical risks for EPICare, we worked with existing clinical and technical teams involved in delivering EIP service provision in five NHS Mental Health Trusts across England (see Table 1 and Supplemental Methods). These Trusts were selected based on ensuring diverse geographical and population coverage across England, including those where predicted need for FEP care has been shown to be high (Table 1) (McDonald et al. 2021). These settings included Birmingham, Nottinghamshire, Greater Manchester, Cambridgeshire and Peterborough, and Camden and Islington (two inner‐city boroughs in North London included in the same Mental Health Trust).

**TABLE 1:** EPICare demonstrator sites population demographics versus England.

Characteristics			
Population characteristics	EPICare^a^	Rest of England	*p*
Ethnic minority population (%)^b^	38.4%	25.3%	< 0.001
Unemployment, median (%)^c^	2.5%	2.5%	0.54
Population density, median (people per km^2^)^d^	4648.9	3390.9	< 0.001
Population density, 1–99 percentile (people per km^2^)^d^	48.3–22553.2	26.5–21320.9	

Pilot demonstrator NHS trusts	Pred. new caseloads (per annum)^e^	Pred. new caseloads (36 months)^e^	% ethnic minorities
Birmingham Women's and Children's	431.2	1293.6	74.8
Nottinghamshire Healthcare	223.2	669.6	43.3
Camden and Islington	148.1	444.3	76.4
Greater Manchester Mental Health	377.0	1131.0	60.6
Cambridgeshire and Peterborough	163.3	489.9	49.2
EPICare	1342.8	4028.4	62.6
England	10949.6	—	50.6

^a^ Five demonstrator Trusts at the time of stakeholder engagement: Birmingham Women's and Childrens; Nottinghamshire Healthcare; Greater Manchester Mental Health, Camden and Islington; Cambridgshire and Peterborough.

^b^ Total population, Census 2021, Table TS021, *X* ^2^‐test *p*‐value.

^c^ Economically active population aged 16+ who reported being unemployed, lower tier local authority level median %, Census 2021, Table TS066, Mann–Whitney *p*‐value.

^d^ Median population density at lower super output area level, Census 2021, Table TS0006, Mann–Whitney *p*‐value.

^e^ Predicted (pred.) caseloads estimated for 2024 from PsyMaptic‐A prediction models (see www.psymaptic.org) (McDonald et al. 2021). Caseloads are new patients treated in EIP services for the first time in each demonstrator site. Caseloads are reported per annum, and for the total duration of the evaluation period (36 months) of EPICare.

We held one‐to‐one meetings with relevant key stakeholders from these five Trusts to provide insights into the routinely collected data, EHR in use, storage and retrieval methods, data quality management, and recording of adherence to the eight NICE standards for FEP treatment (Table 2), as well as mandated outcome measures (see Supplemental Methods), and baseline sociodemographic data. Findings from this process were integrated with Work Package 1 to refine our framework and develop technical infrastructure, governance protocols, and toolkits.

**TABLE 2:** Eight National Institute for Health and Care Excellence (NICE) psychosis standards and NHS England recommended outcome measures.

Item	Explanation
Treatment interventions	
Standard 1: Timely Access to Care^a^	This is the start of EIP treatment which should be ≤ 2 weeks from the date of referral to the start of treatment
Standard 2: CBTp Offer^a^	This is the offer of Cognitive Behavioural Therapy for Psychosis (CBTp)
Standard 3: Offer of family interventions^a^	Family members offered family interventions
Standard 4: Clozapine prescribing^a^	Patients with inadequate response to ≥ 2 antipsychotic drugs offered clozapine
Standard 5: Receipt of supported employment and education programmes^a^	Patients wishing to find or return to work/education offered supported programmes
Standard 6: Physical health checks^a^	Patients have specific comprehensive annual physical health assessments
Standard 7: Physical health interventions^a^	Patients offered combined healthy eating, physical activity programmes and smoking cessation interventions
Standard 8: Carer support^a^	Carers offered carer‐focused education and support programmes
Outcome measures	
Health of the Nation Outcome Scales (HoNOS)^b^	CROM: Safety, substance use, physical health, symptoms, social issues
Process of Recovery Questionnaire (QPR)^b^	PROM: Personal recovery (connectedness, hope, identity, meaning to life, empowerment)
Dialogue^b^, ^c^	PROM: Quality of life, care needs, treatment satisfaction
Goal Based Outcomes (GBO)^c^	PROM: Personal goals
ReQoL‐10^c^	PROM: Personal recovery factors, physical health

Abbreviations: CROM, Clinician Rated Outcome Measure; PROM, Patient Reported Outcome Measure.

^a^ NICE psychosis standards (NICE 2015).

^b^ EIP Service recommended outcome tool. As a minimum, to be used at: initial assessment, 6 months, 12 months, annually, discharge (NHS England 2023).

^c^ Recommended outcome tools for services outside of EIP (NHS England 2023).

### 2.3 WP3: Implementation Evaluation Framework

In this work package, we sought to understand how key stakeholder groups (health informaticians/developers, PPIE representatives, and clinicians/clinical academics) made sense of the EPICare platform in terms of its rationale and purpose, its potential to transform current modes of service delivery, and to identify what stakeholders anticipated as the barriers and drivers to future implementation and uptake. We then sought to understand how such views, as expressed through workshops and participatory methods, contributed to the formative development of the EPICare platform, in anticipation of future implementation. Our approach was informed by well‐established theories and approaches in implementation science, namely the Consolidated Framework for Implementation Research (Damschroder et al. 2022), Normalisation Process Theory (May et al. 2009), and Knowledge‐to‐Action Framework (Graham et al. 2006), that support analysis of how feedback loops in the participatory process shaped the platform's development (see Supplemental Methods for further details).

Data were gathered through workshop observations and semi‐structured interviews. Two independent researchers [JW and TB] observed each workshop to understand how participants engaged, focusing on their knowledge of other health registries and the EPICare platform's role in supporting EIP services. The observations were guided by a semi‐structured observation guide (see Supplemental Methods). In total, a pre‐workshop planning meeting and three workshops were observed over a total of nearly 10 h. Additionally, we interviewed 21 participants from five stakeholder groups (4 patients, 2 carers, and 15 clinical/medical representatives, researchers, and IT developers). These interviews were transcribed and analysed using a framework approach (Gale et al. 2013), where framework themes addressed the stated study objectives (i.e., understanding of the problem EPICare was trying to address, or how it was expected to function in practice).

### 2.4 Patient and Public Involvement: Lived Experience Contributors

PPIE co‐design was embedded from the outset, led by authors with extensive expertise in this field [SA and NC] (see Supplemental Methods). Twelve people with lived experience of psychosis care, though not necessarily of EIP care, from diverse backgrounds were recruited to form the PPIE membership of the stakeholder group, including stakeholders from Cambridgeshire & Peterborough Foundation Trust, Bristol Lived Experience Advisory Panel, and UCL Policy Research Unit PPIE group. Lived experience contributors were reimbursed in line with prevailing NIHR‐recommended rates (www.involve.org.uk).

## 3 Results

### 3.1 WP1: Stakeholder Co‐Design of the EPICare Registry and Dashboard

#### 3.1.1 Stakeholder Characteristics and Work Package Adaptation

Forty stakeholders were initially recruited (13 Early Intervention in Psychosis service professionals, 12 lived experience contributors, 11 other clinical, medical and NHS representatives, two IT specialists, and two academics in mental health epidemiology). During the project we updated the eligibility criteria for the PPIE contributors to require experience of an EIP service in the past 5 years to ensure that the lived experience of Experts by Experience reflected that of modern EIP services rather than older models of psychosis treatment. The second iteration of the PPIE contributor group included 8 members, including two from the previous group. Following feedback from the first stakeholder workshop, we adapted subsequent workshops to include breakout rooms that grouped stakeholders by domain expertise to ensure additional time and space for concentrated discussion according to specialist knowledge. Findings from these groups were brought back to the whole group sessions and summarised by a facilitator in each breakout room.

#### 3.1.2 Registry and Dashboard Data Item Prioritisation

Our first three workshops led to the identification of our adapted MoSCoW data elements, as described below and summarised in Table 3.

**TABLE 3:** EPICare core dataset according to adapted MoSCoW prioritisation.

Must have	Could have	Won't have
Routine baseline demographic data – Dob – Sex – Gender – Ethnicity – Education level – Employment status – Postcode at referral	Carer involvement	Trauma
EIP service	Pathways to care	Family/living status
NCAP audit data – Timely access (first referral, treatment dates (NHS England 2023)) – NICE treatments (NHS England 2023) – NICE outcomes (NHS England 2023)	Medication details	Crisis prevention
Symptoms: Positive, negative, depressive, excitability, cognition	Number of CBT sessions	Continuity of care
	Engagement	Income and welfare support
	Admission to hospital	
	Migration	
	Substance misuse	
	DUP	

##### 3.1.2.1 Must Haves

These included routine baseline sociodemographics (Table 3); the eight NICE psychosis standards collected for the NCAP (Royal College of Psychiatrists 2025); outcome data on personal recovery, quality of life, general health and social functioning. Social and functional recovery were highly valued, with consensus that this could be partially captured through existing measures like HoNOS and routine sociodemographic data. There was also consensus on the need to record longitudinal symptomatology data (positive, negative and cognitive symptoms), seen as a critical, missing clinical measure essential to monitor symptomatic recovery, and to support both individual‐level clinical decision‐making, and population‐level evaluation of equitable and effective care. Further stakeholder consultation in Workshop 4 and WP2‐3 allowed us to consider the feasibility of different reliable and validated instruments for these purposes (i.e., Clinical Global Impression for Schizophrenia (CGI‐Sz), Positive and Negative Symptom Scale (PANSS), Digit Symbol Substitution Task, Symbol‐Symbol Substitution Task).

##### 3.1.2.2 Could Haves

Stakeholders identified several additional elements as desirable but not essential. PPIE representatives, supported by other stakeholders, advocated for including broader social recovery measures (goal‐based PROMs, time use, relationships, family/living arrangements), experiences of trauma, and family/carer‐reported outcome measures. Clinicians and other academic stakeholders identified medication/prescribing data as a “could have” element, as well as number of CBT sessions attended, treatment engagement and hospital admission. Consensus emerged that all these aforementioned items were not “must haves” or “should haves” due to technical and capacity challenges that were identified during knowledge exchange with WP2, thus they were all included as “could haves” (Table 3). Additional “could have” elements were identified (i.e., duration of untreated psychosis, migration status, English as a second language and substance use), however, only the EPICare investigators recognised as having potential future utility. Further, feasibility was limited for measures such as DUP, childhood trauma and medication due to technical limitations in NHS data formatting, data capture or accuracy. Therefore, since they lacked clear consensus during stakeholder consultation, we retained these items on a longlist for potential modular implementation after the initial core “must have” feature set had been implemented on the EPICare platform.

##### 3.1.2.3 Won't Haves

We identified several data elements where there was broad agreement that, while valuable, they would be difficult to capture in a national registry and therefore fell outside of the scope of our immediate prioritisation. Examples included trauma and stressful life events, living arrangements, continuity of care, income, and qualitative data that could capture narrative accounts of psychosis care and recovery. Lived experience contributors particularly desired qualitative input methods in the interest of enriching data, accessibility and providing a means for service users to self‐capture details of care and recovery. Following careful consideration, these means of data collection were deemed impractical within a registry context.

#### 3.1.3 Timing and Frequency of Data Collection

There was consensus that outcome measures should be recorded more frequently than the minimum required frequency for audit purposes (as mandated by the national standard for service users at the time: once at initial assessment, once at another time point (NHS England 2023; Royal College of Psychiatrists 2025)), in line with the changing needs of the patient. Achieving more frequent outcome measurement would also ensure compliance with NHS England guidance on implementing the Access and Waiting Time Standards in EIP care (NHS England 2023). However, clinicians also acknowledged that more frequent capture of PROMs may be difficult to achieve given the balance of engagement and burden for some patients. Consensus opinion suggested a minimum of every 6 months would provide the minimum desirable interval for outcome measure recording, with the frequency of this to be reviewed in the pilot phase.

#### 3.1.4 Functionality of the Clinical Dashboard: Reminders and Notifications

Essential notifications and reminders were identified as useful to clinicians: notifications about the eight NCAP audit standards (aligned to NICE treatment standards for adults with psychosis (NICE 2015; NHS England 2023)), timed in line with national and/or local targets; and notifications about outcome measurement recording (i.e., PROMs and CROMs). Further desirable functionality identified by stakeholders were not prioritised due to likely additional technical, clinical or governance complexity, but retained on a longlist for possible future integration.

#### 3.1.5 Functionality of the Clinical Dashboard: Timing and Frequency

Stakeholders recognised that the timing and frequency of notifications would be highly dependent on the clinician, outcome in question, and time‐sensitivity of the notification (see Supplemental Results for further details). They felt that consideration should be given to clinicians' caseloads, and that notifications should be tailored to specific needs; such flexibility was thought likely to increase uptake. Similarly, given that EIP teams work differently, user‐defined modifications in this regard were also deemed useful.

Finally, a live dashboard detailing service‐ and patient‐level metrics (e.g., against the eight NICE AWT standards) was viewed favourably as a means of getting an immediate sense of how services are operating relative to such standards. PPIE stakeholders additionally expressed a desire for a patient‐facing app, flexible to individual needs, that would allow for greater understanding of their care programme and recovery, and allow patients to directly record and monitor elements of their recovery journey including any PROMs on a frequency of a minimum of 6 months, but with the option to record and track this more regularly, if desired.

### 3.2 WP2: Informatics Architecture, Infrastructure, and Integration: Framework, Protocol and Toolkit

#### 3.2.1 Current Data Collection Within the NHS Trust

Differences in data collection emerged across the five mental health Trusts. Some had mature systems already capturing data routinely; others were less mature with only a subset of measures collected, differences across EIP teams, and manual rather than automated approaches to some elements of data collection.

#### 3.2.2 EHR Used and Data Extraction Options

Different EHR systems were found to be used in the different Trusts (Carenotes, RiO, Paris and SystemOne). Additionally, at the time of stakeholder engagement four of the five Trusts were either in the process of migrating to a different EHR system, had plans to do so in the near future, or were using more than one EHR system. There were also different options for data extraction from the EHR to the dashboard, with some Trusts favouring a particular technology and others offering a choice (e.g., CSV file transfers vs. Power Query to import data straight from the SQL server).

### 3.3 WP3: Implementation Evaluation Framework

Implementation evaluation enabled us to understand how stakeholder groups made sense of the EPICare platform in terms of its rationale, purpose and potential to transform service delivery (Table 4).

**TABLE 4:** Implementation evaluation results: Quotes from stakeholder group participants.

WP3 group	Quote numbers	Contributor	Quote
(1) Health Informaticians/Developers	1	Clinical Academic	“…less than 50% of people with first episode psychosis get into remission and we have a lot of effective treatments, but we don't really know how they're being delivered and the best way for the right person so that's where I view this is going to add a lot of value”
	2	Developer 1	“It will reference the NICE guidelines and it may prompt the clinicians to do particular assessments if they haven't been done on a timely manner or to try a particular intervention if the system detects that would be appropriate for the patient”
	3	Study Investigator	“[EPICare] would allow a service, for example, [City], to know exactly how many people are presented what interventions they've been given from this [set] of NICE recommended interventions, potentially for how long and what impact they've made…we could then see in a real‐world setting whether interventions are actually producing the outcomes they're designed to or whether there's a difficulty in the transition from this idealised clinical trial setting to the clinical application or if this whole population of people that aren't accessing the treatment that we know would be affected potentially would be effective for all sorts of reasons”
	4	Developer 1	“There's really two bits to [EPICare], one is a national registry will be putting together data from lots of different interests about how early intervention psychosis is delivered and what interventions are provided and that data will be collected from the trust IT systems…and the second part is the clinical decision support system which is more of a frontline clinical thing that the clinicians will use to help support them in managing their patients.”
	5	Clinical Academic	“I think setting up a national registry…can, as we know, for lots of disorders…just kind of provides us with the information that we need to improve the…care about when it's delivered how it's delivered”
(2) PPIE Representatives	6	JS, PPIE Contributor #4	“You get the data, hopefully, the data will prove conclusive and people say well such and such treatment works so this is what we're going to change now and we're going to put this treatment in place”
	7	JG, PPIE Contributor #6	“…kind of like a personalised healthcare record to try and make sure that yeah outcome so recovery rates are better identified and observed and that there could be potential solutions for people in terms of decision making, I guess, from clinicians so it's kind of like to be able to kind of aid the clinicians' way of working to make it much more effective and based on data and evidence”
	8	JG, PPIE Contributor #6	“It's kind of those electronic systems that are based purely based for professionals and you know the server machines or patient care never sees that system…and I just wonder whether yeah if you're creating all these kinds of you're creating a system where it would be more beneficial to have the patient carer interact with this system as well”
	9	RS, PPIE Contributor #2	“They're trying to get a programme which will be the same all over the country so that people will have access to a standard treatment”
(3) Clinicians/Clinical Academics	10	JA, Clinical stakeholder #3	“I think setting up a national registry…can, as we know, for lots of disorders…just kind of provides us with the information that we need to improve the…care about when it's delivered how it's delivered, provide evidence that supports increase in funding, development of services”
	11	PH, Clinical stakeholder #8	“I think the first potential value is to have uniformity…not every centre is doing the same thing at the moment”
	12	MB, Clinical stakeholder #2	“[There is] value in terms of highlighting the importance and potential benefits of digitalising healthcare and outcomes and I guess it remains to be seen how personalised those outcomes are going to be”
	13	DR, Clinical stakeholder #4	“If there is an effective flexible user‐friendly menu system that can tie particular problems to particular interventions that it could be really useful the fear is that all it will do is replicate the old fashioned psychiatric…and just repeat what it says in the outdated NICE guidelines which wouldn't be helpful at all. I'm hoping that EPICare will really take things to the next level of measurement and intervention recommendations that could help local clinicians”

#### 3.3.1 Health Informaticians/Developers

The developers articulated four perceived needs for and benefits of a clinical registry for EIP services (see Supplemental Results for further details on this stakeholder group).

The first related to clinical need as manifest in evident disparities in service delivery and in health outcomes (Table 4 quote 1). The second centred on the perceived benefits of the EPICare platform for clinical practice and care delivery (Table 4 quote 2). At the system level, it was suggested that EPICare could reduce variations in service provision by allowing service providers to compare and benchmark current practices and thereby identify and share best practices in service configuration and delivery (Table 4 quote 3).

The third perceived need centred on the technological potential for health informatics to make better use of seemingly under‐utilised routine service data. It was suggested that EIP services, alongside other parts of the NHS, collect data about individual patients, their treatments, and health outcomes, and service‐level data; however, this was difficult to aggregate and analyse (Table 4 quote 4).

The fourth view was that clinical registries had previously been developed and used in other clinical areas, such as stroke care, where they had been successfully used to both appraise and improve services at both the national and local level. It was thought that this approach could be mirrored within EIP services to replicate the types of interventions used in other health services (Table 4 quote 5).

#### 3.3.2 PPIE Representatives

In the PPIE group (see Supplemental Results for further details on this stakeholder group), the EPICare platform was understood primarily in terms of its potential to improve clinical decision‐making and personal care management by making available the up‐to‐date scientific evidence about treatment options and providing evidence to commissioners and other NHS stakeholders about optimal models for EIP services (Table 4 quote 6).

Many participants made sense of the EPICare platform as how it might impact on their own care, or what other service users might expect from a clinical registry in the form of a more personalised record‐keeping, treatment planning, and care management system (Table 4 quote 7).

For many in this group, the EPICare platform was understood more as a personal health record, rather than a national clinical database. There was also an expectation that the EPICare platform should be accessible to service users so that they could enter information directly into the system about, for example, their health and wellbeing or treatment experiences, and that this would then inform better clinical decision‐making and care management (Table 4 quote 8).

Compared with the other stakeholder groups, PPIE contributors demonstrated the most substantial change in their understanding of the EPICare platform through their involvement in the co‐design process. In particular, the workshops appeared to clarify their understanding of how the EPICare platform was similar to clinical registries used in other clinical areas. It was also reported that the EPICare platform reflected a broader agenda to standardise care and reduce variations through providing more consistent evidence (Table 4 quote 9).

#### 3.3.3 Clinicians and Clinical Academics

Amongst clinical stakeholders (which included both clinicians and clinical academics) there was a broader belief that digital informatic systems, such as the EPICare platform, would transform and improve the organisation and delivery of care. Whilst the NCAP had been in place for several years as a retrospective, manualised review of EIP service performance, current processes for measuring service outcomes were viewed as outdated and not utilising new information technologies to provide more timely, comprehensive, prospective and evidence‐based contributions to regional service planning and day‐to‐day clinical decision‐making. Clinical stakeholders were generally familiar with the rationale for clinical audits, and as such the EPICare platform was seen as potentially bringing EIP services up to date in line with other specialities and enhancing the use of existing information systems (Table 4 quote 10). Additional quotes are provided in the Supplemental Results.

Clinical stakeholders shared the view that there was a lack of standardisation in care across the NHS and therefore saw efforts to promote standards through the systematic collection and benchmarking of data as having the potential to promote and raise standards around best practices (Table 4 quote 11).

Some questioned the extent to which the EPICare platform would contribute to highly personalised care and how the clinical dashboard element would function alongside the EPICare core dataset. For these participants, it seemed difficult to comprehend how standardised data collection and the aggregate analysis of this data could inform their practice (Table 4 quote 12).

A small number also questioned whether the EPICare platform would replicate the NCAP, and how the new platform would interface with or replace the existing national audit, again with apprehension about duplication of work (Table 4 quote 13).

Overall, there was much greater alignment between clinical stakeholders' views of the EPICare platform and those of the developers, while PPIE representatives brought additional perspectives and priorities to the discussions. Clinicians were broadly familiar with clinical registries and were supportive of using health information technologies to improve standards across national services and to support day‐to‐day clinical practices.

## 4 Discussion

### 4.1 Principal Findings

This paper reports the main findings from the co‐development stage of our EPICare national LHS for psychosis. We achieved engagement with multiple relevant stakeholder groups, including people with experience of mental health services use, their carers, clinicians, service managers and providers, commissioners and policymakers, academics, industry, and technical and governance specialists within the NHS. WP1 allowed us to engage with stakeholders to derive levels of consensus for essential and desirable data elements that must or could feature in an LHS for psychosis, as well as the optimum frequency and method of collection of those elements. Via a reciprocal and iterative exchange of knowledge with technical developers in WP2, we were able to modify and refine this specification to de‐risk feasibility issues surrounding the technical implementation of the EPICare platform in NHS Trust IT environments. WP3 used implementation science methods to recruit key stakeholders into focus groups and interviews to identify and further derisk possible implementation challenges of deploying the EPICare system from service user, clinician, and provider perspectives. This process allowed us to identify the potential drivers and implementation challenges that may be faced in future stages.

Our co‐designed stakeholder approach allowed us to reach consensus on a core feature set for the EPICare national psychosis registry and integrated dashboard, including core sociodemographic data, NICE psychosis standard data, and symptomatology data as “must have” items. Importantly, this feature set was seen as feasible from technical and governance perspectives at the Trust level. Sociodemographic data, NICE psychosis standard data, and outcome measures (PROMs and CROMs) are already routinely recorded in EIP services, minimising additional burden to core staff. The lack of routinely recorded medication and symptomatology data in EIP services was seen as inhibiting personalised care and local, regional, and national evaluation of what works for whom and the delivery of equitable, effective care in the UK. Where possible the EPICare platform will include resource‐efficient methods of collecting such data longitudinally during EIP care to overcome these challenges. In WP2 we identified potential barriers and facilitators to deploying EPICare in diverse NHS Trusts, serving to “de‐risk” the next stage of this programme. WP3 findings show that a national psychosis registry is highly valued by clinical stakeholders but highlight the need to carefully test its ability to deliver person‐centred care and fully integrate all stakeholders' perspectives.

### 4.2 Identifying and Addressing Implementation Challenges and Opportunities

After introducing the rationale, purpose and proposed way in which the EPICare platform would work as an LHS, several important stakeholder groups understood, supported and saw the opportunities for an interoperable LHS deployed across national EIP services. This suggests that the deployment of a LHS in psychosis care—the first of its kind for any mental health outcome in England—has the potential to be seen as desirable amongst a large group of diverse stakeholders.

Despite this, three key challenges emerged during stakeholder engagement across our three work packages: technical implementation issues, clinical behaviour change, and PPIE representative understanding of the utility of the EPICare platform as an LHS.

First, the primary technical implementation challenge identified in WP2 was concerned with deploying the EPICare platform in Trust IT environments that used different EHR systems and that were considered to be at different levels of maturity. This knowledge allowed us to mitigate and prepare contingency plans for the development and deployment of the EPICare platform to adopt various approaches. To mitigate some of this risk, we established regular communication and engagement with IT and governance teams in each Trust to specify the technical behaviour of the EPICare platform in each EHR and prepare and agree timelines to implementation. As a contingency, particularly with a view to wider national adoption beyond our five Trusts at a later point, we engaged with the provider of the main EHR system that is used in 70% of NHS Mental Health Trusts in England to build the EPICare platform directly into the core EHR.

Second, some clinicians raised concerns about the uptake of the EPICare clinical dashboard and patient‐facing smartphone application in clinical practice, where services, clinicians and patients often have limited capacity to adopt new methods of routine service provision. This knowledge has allowed us to develop plans to overcome this implementation challenge: a brief training program for clinician and patient onboarding, facilitated by a bespoke onboarding video; identification and additional training of an “EPICare champion” in each EIP team to support clinicians using EPICare; single sign‐on methods to lower the barrier for using the clinical dashboard; an intuitive, co‐designed dashboard that identifies overdue treatment measures and PROMs tracking over time to support clinical decision making; and finally, regular visits by the EPICare team to showcase the benefits of using EPICare in routine practice.

Finally, we observed differences in PPIE stakeholders' understanding and agreement of the utility of the EPICare platform as an LHS, and how an LHS could improve patient outcomes, reduce disparities in care and increase service fidelity to national standards. We addressed this by designing additional training and engagement sessions with these stakeholders, and our results suggested this led to the greatest knowledge gain of any stakeholder group. We also formalised lived experience involvement to ensure continued feedback and engagement from people with lived experience in the onward design, implementation and evaluation of the EPICare platform, by establishing a Lived Experience Advisory Group which included PPIE leadership and lived experience in the core EPICare team [NC, SA] and two PPIE members to sit on our Programme Steering Committee. The inclusion of voices from people with lived experience also drove us to design several PPIE activities that were highly desired by people with lived experience of psychosis (co‐design of materials for the EPICare platform and the inclusion of relevant outcome measures and a personal mood diary in the CareLoop EI smartphone app).

### 4.3 Strengths and Limitations

Strengths of this programme development phase of the EPICare platform include the use of an iterative co‐design process leveraging the perspectives of a wide range of key stakeholders who directly or indirectly shape the organisation and delivery of EIP services. By engaging a diverse group of relevant stakeholders, we have ensured that the EPICare platform and clinical dashboard will integrate the features considered most important for providing standardised information on the treated burden of psychosis in the NHS, evaluating real‐world outcomes, identifying priority areas for service improvement, and ensuring equitable, responsive, local resource allocation.

However, current NHS technical, feasibility, information governance and other constraints will limit our ability to include all data elements suggested by stakeholders (such as collecting PROM data more frequently); nevertheless, we consider our core “must‐have” feature set to be a robust starting point that we can build upon over time, depending on need.

### 4.4 Future Directions and National LHS Feasibility

The results of this programme development work will inform the next stages of the EPICare platform rollout. We can now approach these with clear stakeholder co‐designed information to produce an implementation strategy for how to develop and scale EPICare for national benefit. In Stage 2 we will build, pilot and refine the EPICare platform in the five demonstrator Trusts and then Stage 3 will evaluate the effectiveness of the EPICare platform against a series of a priori research questions that seek to understand the real‐world effectiveness of treatments on patient outcomes, disparities in care and outcomes, and the ability of a clinical dashboard to enhance fidelity to national standards of psychosis care.

The EPICare platform will be built (Stage 2) following strict data security standards and governance and considerations for the feasibility of EPICare as a national LHS, such as the costs, cost‐effectiveness, and clinical effectiveness of EPICare to ensure scalability and sustainability at the national level, will be explored during Stage 3 (see Supplemental Discussion).

EPICare itself is also situated alongside other population registries from which the platform has been informed, such as the stroke, CF, and cancer registries in the UK and LHS for psychotic disorders in the United States and Canada (HONE and SARPEP) (discussed in Supplemental Discussion). A national roll‐out of EPICare would complement and build upon the existing UK registries for other health domains, as well as international efforts to provide LHS to optimise EIP care in different jurisdictions.

## 5 Conclusion

We successfully used a co‐designed stakeholder approach to design and develop a LHS for psychosis with a diverse, interdisciplinary group of stakeholders from across academic, clinical and lived experience settings. Through the three work packages, we reached consensus of the “must have” data elements and design of the platform; interrogated and refined the LHS design based on technical implementation feasibility; and identified drivers and implementation challenges from three key groups: clinicians, service users and providers. Through this iterative design based on implementation science methods, we developed a LHS implementation strategy which we will use in Stages 2 and 3 of the EPICare project and may act as a blueprint for the development of future LHS.

## Funding

The EPICare platform is funded in three stages. Stage 1 (stakeholder co‐development) activity was funded by an NIHR Programme Development Grant (NIHR203669). Stage 2 activity (implementation) is funded by the NIHR Mental Health Translational Research Collaboration Mental Health Mission. Stage 3 (evaluation) is funded by an NIHR Programme Grant for Applied Research (NIHR206807). JBK, ML, and IFC were supported by the NIHR Biomedical Research Centre University College London Hospital. IFC is supported by the UCL‐Windsor Fellowship Research Opportunities scholarship. RU and other aspects of this research (i.e., Stage 2) is funded/supported by the NIHR Biomedical Research Centre Oxford Mo Health (NIHR203316). The views expressed are those of the author(s) and not necessarily those of the NIHR or the Department of Health and Social Care. JA is funded by the NIHR Manchester Biomedical Research Centre. All research at the Department of Psychiatry in the University of Cambridge is supported by the NIHR Cambridge Biomedical Research Centre (NIHR203312) and the NIHR Applied Research Collaboration East of England. The views expressed in this publication are those of the authors and not necessarily those of the NHS, the NIHR, or the Department of Health.

## Ethics Statement

The EPICare platform was reviewed and granted full approval by the Health Research Authority on November 8, 2021 (Ref: 306234). The EPICare programme was reviewed and approved by the East Midlands—Derby Research Ethics Committee on 08/06/2024 (REC reference 24/EM/0151; IRAS ID: 334905).

## Conflicts of Interest

R.U. reports speaker fees from Sunovion, Springer Healthcare, Otsuka, and consultancy with Bristol Myers Squibb. G.K.M. has received consultancy fees from IESO and consults for Bristol Myers Squibb. M.K. has received consultancy and speaker fees from Teva. J.A. is a shareholder and director of CareLoop Health Ltd.; President of the Institute of Health Records and Information Management (non‐pecuniary); and has received consultancy fees from Academic Health Solutions Ltd. P.W. is Chief Operating Officer, shareholder and director of CareLoop Health Ltd. and receives consultancy fees from the Wellcome Trust.

## Supporting information


**Data S1:** Supporting Information.

## Data Availability

The data that supports the findings of this study is available in the main text and Supporting Information of this article.
