# Supplementary material for: A Stakeholder‐Informed Approach to the Development and Implementation of National Digital Registry and Learning Healthcare System (LHS) for Psychosis: Findings From the EPICare Platform
Source: Early Interv Psychiatry. 2026 Sep 21;20(9):e70264. doi: 10.1111/eip.70264 (PMC13591294; doi:10.1111/eip.70264)
Supplement: Supplementary file 1 [file EIP-20-0-s001.docx]

**SUPPLEMENTAL METHODS**

**Work package 1: Stakeholder recruitment - Further information**

We conducted a stakeholder mapping exercise to identify stakeholders with relevant expertise and experience. Based on co-investigator expert knowledge, we first identified key domains where relevant knowledge was likely to be located (clinical, academic, policy, lived experience, etc). Next, we mapped potential relevant stakeholders using our co-investigator expert knowledge, snowball sampling and word-of-mouth. This ensured breadth of coverage across stakeholder interest groups, geographical coverage and participant knowledge. Clinical stakeholders were required to be working within an EIP service and were additionally approached by the lead clinical academic for EIP services in each of our study sites. Patient and public involvement (PPIE) contributors were recruited using social media (recruitment materials were posted on Twitter and circulated via team members’ professional accounts) and via established networks (existing links held by team members in Bristol, Birmingham and London were used to circulate news of the involvement opportunity to relevant groups). PPIE contributors were required to: be aged 18 or older, have received care through EIP services within the past 5 years or have experience of supporting someone with this experience and have an interest in the development of existing EIP services.

**Work package 2: Further information**

The initial Trusts which agreed to participate in stakeholder co-development were Birmingham Women’s and Children’s NHS Mental Health Trust (with mental health service provision, including EIP services later transferred to Birmingham & Solihull Mental Health Foundation Trust in 2025), Camden and Islington NHS Foundation Trust (which later merged with Barnet, Enfield and Haringey NHS Foundation Trust to form the North London Foundation Trust in 2024), Greater Manchester Mental Health Trust, Nottinghamshire Healthcare Foundation Trust, and Cambridgeshire and Peterborough Foundation Trust.

The NHS England Access and Waiting Time standard, first published in 2016, with revisions in 2020 and 2023, mandated the collection of various clinician- and patient-reported outcome measures (CROMs and PROMs, respectively) in EIP services. These included: HoNOS (Health of the Nation Outcome Scales; a PROM), DIALOG (a PROM), Goal Based Outcomes (GBO; a PROM), Recovering Quality of life (ReQOL; a PROM), and QPR (Questionnaire about the Process of Recovery; a PROM).

**Work Package 3: Implementation evaluation framework – Further details**

Our approach was informed by well-established theories and approaches in implementation science to focus on how stakeholders’ understanding of challenges in early psychosis care, the potential impact of the registry and clinician dashboard on service improvement, expectations for intervention use in practice, and experiences with the co-design process. An important feature of the study design was that stakeholders were invited to consider the anticipated or possible implementation and impact of the EPICare platform so that these insights could then inform its on-going and future development. As a type of implementation research this offers an ‘upstream’ analysis that can trace the antecedent factors that might shape future downstream spread and adoption. At the same time, it examines how the views of stakeholders then shaped the ongoing and formative development of the EPICare platform as part of the participatory design. As such, it allows analysis of the ways in which feedback loops in the participatory process shaped ongoing development, especially with respect to consideration of and forward planning for downstream implementation.

The observations were guided by a semi-structured observation topic guide, as detailed below:

*Prompts for observation record:*

- Composition of Group
- Structuring, facilitation, organisation
- Outline of exchange and discussion
- Presentation of EPICare: purpose, rationale, evidence, framing, advantage (style, approach)
- Responses to EPICare: cohesion, clarifications, agreement, disagreement, alternate (to what extent to different views lead to different viewpoint)
- Issues raised: owners, confidential, risk, operational issues, priorities
- How workshop activities were presented and how this shape interaction
- Balance of exchange (dominant/marginal/silenced, etc)
- How are disagreements manifest and managed

**Patient and Public Involvement: Lived experience contributors – Further details**

PPIE co-design was embedded from the outset, led by authors with extensive expertise in this field [SA, NC]; Dr Sophie Allan is a member of the study team with expertise by experience and Niyah Campbell is the Facilitation Lead for the University of Birmingham Youth Advisory Group (YAG) at the Institute for Mental Health. Lived experience stakeholders were recruited by means of an EPICare PPIE Invitation Flier and Information Sheet. Lived experience contributors were reimbursed in line with prevailing NIHR-recommend rates (www.involve.org.uk), which equated to £25 per hour of participation at the time of involvement.

**SUPPLEMENTAL RESULTS**

**Work Package 1: Stakeholder co-design of the EPICare registry and dashboard: Further details**

*Functionality of the clinical dashboard: reminders and notifications*

Desirable functions of the EPICare dashboard reminders and notifications that were retained on a longlist for possible future integration included: notifications about patient engagement (e.g., whether they were seen in the past month); reminders of key Mental Health Act dates (e.g., tribunal dates, Section 117 reviews); a countdown to discharge upon three years of EIP care; reminders of referrals to be made to other services (e.g., Child and Adolescent Mental Health Services); prompts to follow up with a patient post-discharge or disengagement; notifications about changes in symptom and/or functioning scores; notifications when a patient meets exclusion criteria for EIP services at baseline; and notifications based on a predictive model, or an embedded Lester-type tool (Perry et al., 2023).

*Functionality of the clinical dashboard: timing and frequency – Further details*

Stakeholders felt that clinician-facing notifications should be outcome- and time-dependent, in that some could be triggered at structured time points (e.g., reminders to offer CBT); whereas real-time notifications would be necessary for risk- or crisis-related factors, such as severely deteriorating outcome measurements and more time-sensitive factors such as Community Treatment Orders. For less time-critical notifications or those not immediately actionable, the ability to mute, snooze, or switch off reminders was widely desired, although with justification (e.g., patient already on CBT waiting list or patient ineligible for Clozapine), such that outstanding notifications were eventually addressed. Stakeholders believed that both pop-up notifications or email reminders may get ignored if too frequent, and that a visible permanent feature (e.g., a color-coded red-amber-green [RAG] rating) may be useful to address particularly important notifications. An alert tree was also thought to be potentially useful in helping guide clinicians through nested notifications, as a self-audit of potentially actionable items.

**Work Package 3: Implementation evaluation framework: Further details**

*Stakeholder group: Health informaticians/Developers*

This group comprised one of the study’s co-principal investigators, a clinical academic and two health informatic specialists who led the overarching research programme, conceptual development and preliminary configuration of EPICare. As might be expected, this group had an advanced understanding of the problems faced in EIP services, together with a developed appreciation of the potential value that EPICare could have in providing an interoperable national clinical registry via its core dataset, as well as its ability to provide real-time, actionable insights to inform evidence-based diagnosis and treatment via its clinical dashboard.

*Stakeholder group: PPIE representatives*

This stakeholder group included people with a lived experience of psychosis within the past five years and people with experience of providing care and/or support to someone with a psychosis diagnosis. In comparison to other stakeholder groups, the PPIE stakeholders had differing familiarity and opportunity for engagement with a digital psychosis platform. Participation in the co-design workshops provided the potential to share and develop their understanding of such a platform and how it could contribute to care services.

*Additional stakeholder quotes*

Here we provide additional quotes from stakeholders to support the themes that emerged in this work package.

*Clinicians/Clinical Academics*

Clinical stakeholders shared a broadly common view of EPICare as a type of digital ‘platform’ to routinely and systematically gather data that could be used to improve the organisation of care against national standards and inform clinical decision-making based on best evidence.

[MB, Clinical stakeholder #2]: *So my understanding is that it's trying to automate [and] digitalise the collection of outcome measures and various data that we routinely collect [in EIP] and that its going to try and extract that data digitally from existing clinical and care records and it will then be analysing that data and benchmarking against...certain parameters and standards to then give feedback to and services and conditions on the ground and make recommendations...and then influence the outcome of that care and importantly patient outcomes in a positive way*

For many, the ability to function at both national and local levels was especially positive:

[DR, Clinical stakeholder #4]: *the EPICare case registries [will] identify cases so that outcomes can be tracked now [on] a national level and the clinicians can be helped locally with advice about what are the best treatments for patients, so that's supposed to be the aim*

[AH, Clinical stakeholder #1]: *[it] is not just about collecting data but how is that going to give value to the [clinicians] and the patients so that motivates people to do things*

A small number of clinical stakeholders described a type of balancing act between maintaining standards and standardisation and, at the same time, providing more person-centred care based on timely evidence:

[CG, Clinical stakeholder #5]: *if we can have that person-centred approach but standardisation around the package that we're offering...so important...and I think it's great if we can get technology on board to make lives easier*

It was common for clinical stakeholders to describe the EPICare platform as ‘giving’ information to inform decision-making, but there were also uncertainties about how data would be collected, how notifications and information would be communicated, and whether it would add to or alleviate administrative burden. Following participation in the co-design workshops, it remained difficult for clinicians to comprehend the precise ease of use of the EPICare platform in day-to-day practice. Many were concerned about the additional time demands of making the system function as expected.

[CG, Clinical stakeholder #5]: *I think it's great if we can get technology on board to make lives easier, I would hate for it to be an additional piece of work for clinical staff, I hope that one would feed into another system*

A further area of ambiguity for clinical stakeholders was the scope and scale of the EPICare platform relative to other audits. Reflecting on the specification activities within the workshops, they welcomed a comprehensive range of measures and data sources to inform better decision-making but also recognised the demands of collating and inputting data and the current limits of NHS information systems. These issues played out in the workshops, where stakeholders deliberated the range of measures to be included in the registry:

[PH, Clinical stakeholder #8]: *I think the challenge is to know how many things that you'd like to do are realistic and how you prioritise that list of desirable but not essential and…my own prejudice is I think markers of cognition and some biological markers have got the greatest traction at the moment*

Following participation in the workshops, clinical stakeholders seemed more concerned about the practical utilisation and operation of the EPICare platform, and whether automated notifications would become an overwhelming and, in time, ignored feature of the system.

**SUPPLEMENTAL DISCUSSION**

**Future Directions and National LHS feasibility – Further details**

*Data security and governance*

Privacy safeguards and high-security, reliable methods of data collation and transfers will be employed by ensuring that no personal data will be handled by the EPICare platform (data will be pseudonymised and stored in a secure Trusted Research Environment). High importance will be placed on information and data security throughout the build and refinement process. During this stage, we will also identify and fix potential issues in data collection (e.g. differences in how Trusts record or measure data) and missingness to reduce such impacting data output and effectiveness evaluations conducted in Stage 3.

*Feasibility of EPICare as a national LHS*

We will interrogate the LHS and output data to understand whether the EPICare LHS: (i) can provide population-level insights into psychosis outcomes and care; (ii) has external validity; (iii) implementation improves fidelity to quality standards, care and clinical outcomes; and (iv) is cost-effective. Stage 3 also offers an opportunity for changes to be made to EPICare if required due to clinical- or cost-effectiveness first within pilot Trusts before a national roll-out.

Within the UK, existing population registries (i.e. the stroke, cystic fibrosis (CF) and cancer registries), have helped to inform our approach to EPICare. While they have all made great impacts on improving outcomes (Heuschmann et al., 2008; Kelsey et al., 2020; Stephan et al., 2020; Taylor-Robinson et al., 2018), there is heterogeneity in their designs and data; the cancer registry is national (England), whereas the stroke and CF registries additionally collect data from Wales and Northern Ireland and the CF registry also includes Scotland (Henson et al., 2020; *Sentinel Stroke National Audit Programme (SSNAP)*, n.d.; Taylor-Robinson et al., 2018).

Outside of the UK, LHS for psychotic disorders have also been developed. HONE (Health Outcomes Network and Education), an informatics platform LHS based in Connecticut, United States, and SARPEP (Système Apprenant Rapide pour les Programmes de Premiers Épisodes Psychotiques), a LHS in Quebec, Canada. Both were designed and developed to support and interrogate effectiveness of EIP services. Similarly to EPICare, HONE and SARPEP were also designed and built across an iterative process with involvement from a range of stakeholders. HONE differs from EPICare in the approach to integration and data reporting; while EPICare aims to be integrated into existing NHS systems, HONE is built to be a standalone system which provides population-level (Connecticut) data collected from all enrolled EIP clinics (Mathis et al., 2025). Conversely, SARPEP was built using an existing open-access platform (REDCap digital platform) and requires participating EIP teams to upload data directly every 4 months (Ferrari et al., 2022). Clinics using SARPEP receive clinic-level data on each of the EIP indicators and, similarly to HONE, can compare their results with aggregated results from all EIP clinics enrolled. SARPEP also collects patient reports of service satisfaction which are fed back to the individual EIS teams. One of the main differences between EPICare and SARPEP is the manual data collection process SARPEP employs.

**SUPPLEMENTARY REFERENCES**

Ferrari, M., Iyer, S., LeBlanc, A., Roy, M.-A., & Abdel-Baki, A. (2022). A Rapid-Learning Health System to Support Implementation of Early Intervention Services for Psychosis in Quebec, Canada: Protocol. *JMIR Research Protocols*, *11*(7), e37346. https://doi.org/10.2196/37346

Henson, K. E., Elliss-Brookes, L., Coupland, V. H., Payne, E., Vernon, S., Rous, B., & Rashbass, J. (2020). Data Resource Profile: National Cancer Registration Dataset in England. *International Journal of Epidemiology*, *49*(1), 16–16h. https://doi.org/10.1093/ije/dyz076

Heuschmann, P. U., Grieve, A. P., Toschke, A. M., Rudd, A. G., & Wolfe, C. D. A. (2008). Ethnic group disparities in 10-year trends in stroke incidence and vascular risk factors: The South London Stroke Register (SLSR). *Stroke*, *39*(8), 2204–2210. https://doi.org/10.1161/STROKEAHA.107.507285

Kelsey, E. A., Njeru, J. W., Chaudhry, R., Fischer, K. M., Schroeder, D. R., & Croghan, I. T. (2020). Understanding User Acceptance of Clinical Decision Support Systems to Promote Increased Cancer Screening Rates in a Primary Care Practice. *Journal of Primary Care & Community Health*, *11*. https://doi.org/10.1177/2150132720958832

Mathis, W. S., Gibbs-Dean, T., Cahill, J. D., & Srihari, V. H. (2025). HONE: A learning health system platform for advancing early intervention in first episode psychosis. *Schizophrenia Research*, *280*, 1–9. https://doi.org/10.1016/j.schres.2025.04.009

Perry, B., Holt, R., Chew-Graham, C., Tiffin, E., French, P., Pratt, P., Byrne, P., & Shiers, D. (2023). *Positive Cardiometabolic Health Resource: An Intervention Framework for People Experiencing Psychosis and Schizophrenia. 2023 Update (with acknowledgement to the late Helen Lester for her contribution to the original 2012 version)*. Royal College of Psychiatrists. https://www.rcpsych.ac.uk/docs/default-source/improving-care/ccqi/national-clinical-audits/ncap-library/eip-2024/ncap-lester-tool-intervention-framework.pdf?sfvrsn=21e45dbd_23#:~:text=This%20resource%20aligns%20with%20the,individuals%20with%20severe%20mental%20illness.

*Sentinel Stroke National Audit Programme (SSNAP)*. (n.d.). Healthcare Quality Improvement Partnership (HQIP). Retrieved 13 July 2026, from https://www.hqip.org.uk/our-programmes/sentinel-stroke-national-audit-programme-ssnap/

Stephan, B. C. M., Siervo, M., & Brayne, C. (2020). How can population-based studies best be utilized to reduce the global impact of dementia? Recommendations for researchers, funders, and policymakers. *Alzheimer’s & Dementia : The Journal of the Alzheimer’s Association*, *16*(10), 1448–1456. https://doi.org/10.1002/ALZ.12127

Taylor-Robinson, D., Archangelidi, O., Carr, S. B., Cosgriff, R., Gunn, E., Keogh, R. H., MacDougall, A., Newsome, S., Schlüter, D. K., Stanojevic, S., Bilton, D., & the CF-EpinNet collaboration. (2018). Data Resource Profile: The UK Cystic Fibrosis Registry. *International Journal of Epidemiology*, *47*(1), 9–10e. https://doi.org/10.1093/ije/dyx196
